# Functionalized Tyrosinase-Lignin Nanoparticles as Sustainable Catalysts for the Oxidation of Phenols

**DOI:** 10.3390/nano8060438

**Published:** 2018-06-15

**Authors:** Eliana Capecchi, Davide Piccinino, Ines Delfino, Paolo Bollella, Riccarda Antiochia, Raffaele Saladino

**Affiliations:** 1 Department of Biological and Ecological Sciences, University of Tuscia, Via S. Camillo de Lellis snc, 01100 Viterbo, Italy; eliana.capecchi@studenti.unitus.it (E.C.); d.piccinino@unitus.it (D.P.); delfino@unitus.it (I.D.); 2 Department of Chemistry and Drug Technologies, Sapienza University of Rome P.le Aldo Moro 5, 00185 Rome, Italy; paolo.bollella@uniroma1.it (P.B.); riccarda.antiochia@uniroma1.it (R.A.)

**Keywords:** lignin nanoparticles, tyrosinase, sustainable catalyst, oxidation of phenols

## Abstract

Sustainable catalysts for the oxidation of phenol derivatives under environmentally friendly conditions were prepared by the functionalization of lignin nanoparticles with tyrosinase. Lignin, the most abundant polyphenol in nature, is the main byproduct in the pulp and paper manufacturing industry and biorefinery. Tyrosinase has been immobilized by direct adsorption, encapsulation, and layer-by-layer deposition, with or without glutaraldehyde reticulation. Lignin nanoparticles were found to be stable to the tyrosinase activity. After the enzyme immobilization, they showed a moderate to high catalytic effect in the synthesis of catechol derivatives, with the efficacy of the catalyst being dependent on the specific immobilization procedures.

## 1. Introduction

It is assumed that renewable materials might play a key role in Circular Economy due to their unique environmentally benign properties [1]. Lignin, nature’s dominant renewable polyphenol, is produced in quantities of over fifty million tons per year [2] as a byproduct of pulp and paper manufacture, and of biorefinery industries [3]. Lignin byproducts are low cost materials that perform as well as petroleum-derived counterparts, showing beneficial properties associated with their aromatic character, such as antioxidant activity [4,5], UV-shielding protection [6,7], anticorrosion effects [8], and electrochemical responsiveness in both charge storage composites [9] and electrochemical sensors [10]. For this reason, lignin is emerging as a new and inexpensive raw material for the preparation of nanocapsules with increased chemical and physical properties [11,12,13,14], due to the direct consequence of “size-effect” phenomena [15]. These nanoparticles can be, in principle, useful polyphenol-based platforms for the immobilization of enzymes [16,17]. The supramolecular interaction between lignin and proteins has been studied in detail, identifying lignin-binding peptide sequences of affinity [18], and analyzing the role that conformational changes of the polymer play in the molecular recognition process [19]. The folding structure of the protein, associated with the prevalence of Coulombic forces in non-ionic medium with respect to hydrogen bonding and hydrophobic polar type interactions, emerged as crucial parameters in the self-assembly of proteins on the lignin surface [20]. Moreover, lignin showed a high boosting activity towards copper oxidative enzymes, such as Lytic Polysaccharide MonoOxygenases (LPMOs), through long-range electron transfer mechanisms between the aromatic moieties and the metal atoms in the active site of the enzyme [21]. In this context, tyrosinase (EC 1.14.18.1) was shown to further improve the LPMOs oriented boosting activity of lignin [22], performing the *ortho*-hydroxylation of phenol moieties to corresponding low redox-potential catechol derivatives (1,2-benzene diols; monophenolase activity) [23]. Thus, lignin and tyrosinase cooperate in synergy to realize efficient oxidative processes. Tyrosinase has been widely studied because of its relevance in several applied areas [24,25], showing better performances in industrial applications when used in the immobilized form, as a consequence of its reusability, enhanced stability, and easier purification procedures [26]. In recent years, we described the preparation of heterogeneous catalysts for the synthesis of bioactive catechol derivatives by the immobilization of tyrosinase on microcapsules of the epoxy resin Eupergit [27,28], and as an alternative, by Layer-by-layer coating of the enzyme on Multi Walled Carbon Nanotubes (MWCNTs) [29]. These catalysts provided a high catalytic efficiency in the synthesis of neuroactive 3,4-dihydroxyphenylalanine (DOPA)-containing peptides [30,31], and of lipophilic catechols with broad spectrum antiviral activity against DNA and RNA viruses [32,33,34]. As a general trend, nanostructured catalysts showed greater activity, storage life, and reusability than the microcapsule counterparts [35]. Here, we describe for the first time the immobilization of tyrosinase (T3824, multimeric enzyme with H2L2-type structure) [36] in nanoparticles of organosolv lignin (OL) [37] by means of four different procedures, namely: (i) encapsulation; (ii) surface adsorption; (iii) Layer-by-layer (LbL) coating; and (iv) LbL coating in the presence of bovine serum albumin (BSA) and glutaraldehyde (GA). In contrast to native lignin and other technical lignins, OL has a relatively low molecular weight and a distinct solubility in organic solvents [38]. The novel catalysts have been characterized regarding their structural and kinetic properties, and they have been successively applied for the oxidation of selected phenols to corresponding bioactive catechols. Notably, lignin-based tyrosinase catalysts prepared by LbL assembly showed activity and kinetic parameters of the same order of magnitude, or higher than, those previously reported for catalysts based on the use of traditional inorganic supports.

## 2. Materials and Methods

### 2.1. Materials

Organosolv lignin (OL) was purchased from Chemical Point (Oberhaching, Germany). Mushroom tyrosinase (1000 U/mg), bovine serum albumin (BSA), glutaraldehyde (GA), poly (diallyldimethylammonium chloride) (PDDA, 20% *v*/*v* water solution), 2-chloro-4,4,5,5-tetramethyl-1,3,2-dioxaphopholane, chrome (III) acetylacetonate, deuterated chloroform (CDCl_3_), ascorbic acid, Bradford reagent, l-tyrosine, *para*-cresol, tyrosol, 4-hydroxyphenylacetic acid, 3-(4-hydroxyphenyl)propionic acid, catechol, potassium chloride (KCl), and organic solvents were purchased from Sigma–Aldrich (St. Louis, MO, USA), and were used without any further purification. All experiments were done in triplicate using native and immobilized tyrosinase in Phosphate buffer saline (PBS).

### 2.2. Reactivity of Tyrosinase with Organosolv Lignin

An appropriate amount of OL (70 mg) was treated with tyrosinase (1000 U) in PBS (5.0 mL, 0.1 M; pH 7) under dioxygen atmosphere at room temperature for 24 h. This mixture was acidified (pH 3, 1.0 M HCl) and successively centrifuged (6000 rpm, 20 min) to remove the supernatant. The residue was rinsed with deionized water and then freeze-dried. The qualitative and quantitative analysis of alcoholic, acidic, and phenolic OH groups in the residual crude after tyrosinase treatment was determined by quantitative ^31^P-NMR analysis [39]. Typically, the lignin sample (10 mg) was dissolved in pyridine/CDCl_3_ (300 µL; 1.6/1.0 *v*/*v*), followed by the addition of chrome (III) acetylacetonate solution (50 mL, 11.4 mg/mL) as a relaxing agent. Then, the phosphitylation reagent 2-chloro-4,4,5,5-tetramethyl-1,3,2-dioxaphopholane (200 µL) was added under magnetic stirring and gentle heating at 45 °C for 2 h. NMR analysis was performed in the presence of cholesterol as an internal standard on a Bruker 400 MHz apparatus (Billerica, MA, USA). Gel Permeation Chromatography (GPC) analysis of OL was carried out following the procedure of acetobromination [40]. Briefly, an appropriate amount of lignin sample (10 mg) was suspended in acetic glacial/acetyl bromide mixture (2.5 mL of 92:8 *v*/*v*) and stirred at room temperature. After 2 h, the solvent was evaporated under vacuum and the residue was dissolved in THF (5 mL). The GPC analysis was performed using a Shimadzu LC 20AT liquid chromatograph (Kyoto, Japan) with an SPD M2OA ultraviolet diode array (UV) detector at 280 nm.

### 2.3. Preparation of Catalyst ***I***

The preparation of catalyst **I** required the encapsulation of tyrosinase within the OL nanoparticles. Briefly, an appropriate amount of tyrosinase (3.0 mg, 900 U/mg) was added to a solution of OL in THF (2.0 mg/mL, 7.5 mL) previously filtered by a 0.45 µm filter membrane (PALL, Port, Washington, NY, USA). The solution obtained was dialyzed against deionized MilliQ water (SIEM, Bologna, Italy) (500 mL) for 24 h at 25 °C under slow magnetic stirring (STUART, Staffordshire, UK), using a Spectrapore™ dialysis membrane (3.5 kD MWCO) (SPECTRUM, Dallas, TX, USA). Catalyst **I** was recovered by centrifugation (20 min at 6000 rpm) (HETTICH, Tuttlingen, Germany) and the surnatant was used for the calculation of the activity parameters. The catalyst was washed several times with PBS in order to ensure the complete removal of unbounded tyrosinase (as evaluated by the Bradford method).

### 2.4. Preparation of Catalyst ***II***

Catalyst **II** was prepared by the direct adsorption of tyrosinase on preformed OL nanoparticles prepared by the dialysis protocol [11]. Briefly, OL solution (THF, 2 mg/mL) previously filtered by a 0.45 µm filter membrane, was dialyzed against deionized water for 24 h at 25 °C with slow magnetic stirring, using a Spectrapore™ dialysis membrane (3.5 kD MWCO). Finally, the OL nanoparticles obtained were centrifuged (20 min at 6000 rpm), washed with Milli-Q water, and lyophilized. The appropriate amount of tyrosinase (3.0 mg, 900 U/mg), dissolved in sodium phosphate buffer PBS (3.0 mL, 0.1 M; pH 7), was added to a suspension of OL nanoparticles (15 mg) in PBS (15 mL) under orbital shaking at room temperature for 24 h. Catalyst **II** was recovered by centrifugation (20 min at 6000 rpm) and the surnatant was used for the calculation of activity parameters. The catalyst was washed several times with PBS in order to ensure the complete removal of unbounded tyrosinase (as evaluated by the Bradford method).

### 2.5. Preparation of Catalyst ***III***

The OL nanoparticles were coated with PDDA applying the LbL procedure [41]. Briefly, a suspension (15 mL) of OL nanocapsules in MilliQ water (1 mg/mL, pH 4.2) was added dropwise to PDDA solution (1 mg/mL, Milli-Q water) under slow magnetic stirring. After 2 h, the final suspension was centrifuged in order to recover OL-PDDA nanoparticles followed by washing with Milli-Q water and was then lyophilized. Tyrosinase (3.0 mg, 900 U/mg) was added to a suspension of OL-PDDA nanoparticles (15 mg) in PBS (15 mL) under orbital shaking at room temperature for 24 h. Catalyst **III** was recovered by centrifugation (20 min at 6000 rpm) and the surnatant was used for the calculation of the activity parameters. The catalyst was washed several times with PBS in order to ensure the complete removal of unbounded tyrosinase (as evaluated by the Bradford method).

### 2.6. Preparation of Catalyst ***IV***

The OL-PDDA nanoparticles were prepared as described above and then used in order to immobilize BSA and tyrosinase by crosslinking with GA. Briefly, tyrosinase (300 μL, 3.4 mg/mL in PBS) and BSA (1.5 mL, 2.0 mg/mL) were added to OL-PDDA nanoparticles suspension (3.5 mg/mL, PBS 1.4 mL), and the mixture was shaken for 30 min at room temperature. Finally, GA (150 µL, 2.5% *v*/*v* water solution) was added and the mixture was shaken again at room temperature for 30 min and then at 4 °C overnight. Catalyst **IV** was recovered by centrifugation (20 min at 6000 rpm) and the surnatant was used for the calculation of activity parameters. The catalyst was washed several times with PBS in order to ensure the complete removal of unbounded tyrosinase (as evaluated by the Bradford method).

### 2.7. SEM Characterization

For Scanning Electron Microscopy (SEM), catalysts (**I**–**IV**) were fixed with 3% GA in 0.1 M cacodylate buffer (pH 7.2), washed, and post-fixed in OsO_4_ (1.0% in weight) in the same buffer at room temperature. The samples were dehydrated in ethanol series before the analysis, air dried, and sputter-coated with gold in a Balzers MED 010 unit (Balzers, Liechtenstein). The observation was made by a JEOL JSM 6010LA electron microscope (Tokyo, Japan).

### 2.8. DLS and Zeta Potential

Measurements of particle size and Zeta potential were performed on freshly prepared aqueous suspensions (pH = 7) of OL nanoparticles and catalysts **I**–**IV** were dispersed in water by dynamic light scattering (DLS) using a Zetasizer Nano ZS (Malvern Instruments, Malvern, UK) apparatus, equipped with an He-Ne laser (633 nm, fixed scattering angle of 173°, 25 °C). Prior to perform size measurements, the suspensions were filtered with a 0.45 µm filter membrane. Measurements were performed in triplicate at room temperature (T = 25 °C).

### 2.9. Determination of Activity Parameters

The activity parameters of the native and immobilized enzyme were determined by measuring the tyrosinase catalyzed oxidation of l-tyrosine by the dopachrome method. As a general procedure, the reaction was started by adding l-tyrosine (0.83 mM) to PBS solution (67 mM, pH 7) containing the appropriate amounts of the native enzyme (from 45 to 100 units), and as an alternative, of catalysts **I**–**IV** (from 0.045 to 0.1 mg). The initial rates were measured in a 3.0 mL reaction cuvette as the linear increase of the optical density (475 nm) at 25 °C under magnetic stirring. One unit of enzyme activity was defined as the increase in absorbance of 0.001 unit per minute at pH 7 and 25 °C. Spectrophotometric data were analyzed with Cary WinUV (AGILENT, Santa Clara, CA, USA). All experiments were conducted in triplicate using free and immobilized tyrosinase.

### 2.10. Determination of the Kinetic Constants

Kinetic parameters, K_m_, V_max_, and V_max_/K_m_, were determined spectrophotometrically by measuring the enzyme activity at different concentrations of l-Tyrosine (0.33–0.10 mM) and by plotting data in Lineweaver-Burk. All experiments were conducted in triplicate using native and immobilized tyrosinase. Reactions were carried out by means of the same procedure as for the activity assay and measuring absorbance at 475 nm, as described above.

### 2.11. Electrochemical Characterization

Cyclic voltammetry experiments were performed by using a PGSAT204N potentiostat (Eco Chemie, Utrecht, The Netherlands) controlled by Nova 2.1 software (Eco Chemie, Utrecht, The Netherlands) with a conventional three-electrodes electrochemical cell equipped with a modified glassy carbon (GC) electrode as the working electrode (d = 3 mm, Cat. 6.1204.300, Metrohm, Herisau, Switzerland), in addition to a saturated calomel electrode (SCE, 244 mV vs. NHE, Cat. 303/SCG/12, AMEL, Milano, Italy) and a glassy carbon rod electrode (d = 2 mm, Cat. 6.1241.020, Metrohm, Herisau, Switzerland), as the reference and counter electrodes, respectively. All electrochemical measurements were performed in 50 mM PBS buffer pH 6.5 plus 100 mM KCl containing 250 µM cathecol, used as an electrochemical mediator [42]. GC electrodes (d = 3 mm, Cat. 6.1204.300, Metrohm, Herisau, Switzerland) were polished with alumina slurries (Al_2_O_3_, particle size of 0.3 and 0.05 μm) on cloth pads wet with Milli-Q water, thoroughly rinsed with Milli-Q water, and further sonicated for 5 min between each polishing step. For the bio-modification, GC electrodes were modified by drop-casting 5 µL of sample OL nanoparticles (2 mg/mL), catalyst **I** (2 mg/mL), catalyst **II** (2 mg/mL), catalyst **III** (2 mg/mL), and catalyst **IV** (2 mg/mL), respectively. For each different modification, three replicates have been performed.

### 2.12. General Procedure for the Oxidation of Phenols

As a general procedure, phenols **1**–**4** (0.02 mmol), tyrosinase (540 Units), and ascorbic acid (2 equiv), were placed in PBS 5.0 mL (0.1 M) at 25 °C for 24 h, and the mixture was stirred under O_2_ atmosphere. In agreement with previous optimization studies reported in the literature, the experiments have been performed at optimal pH 7.0 [43]. Oxidations were performed using homogeneous and heterogeneous conditions. The reactions were monitored by thin layer chromatography (TLC, n-hexane/EtOAc = 2.0:1.0). After the disappearance of the substrate, the reaction mixture was acidified with a solution of HCl 2.0 M and extracted twice with EtOAc (2 × 10 mL). The organic extracts were treated with a saturated solution of NaCl and dried over anhydrous Na_2_SO_4_, then filtered and concentrated under vacuum. In the case of the immobilized enzyme, catalysts **I**–**IV** were recovered by centrifugation (6000 rpm) for 20 min and the solution was subjected to the same work up as described above. The residue was treated with pyridine, HMDS, and TMCS (HMDS–TMCS, 2:1 *v*/*v*) under vigorous stirring at room temperature for 2 h. Products were identified by gas-chromatography associated with mass-spectrometry analysis (GC-MS). The analyses were performed by 450 GC-320 MS apparatus (VARIAN, Palo Alto, CA, USA) using a VF-5MS column and an isothermal temperature profile of 100 °C for 2 min, followed by a 10 °C/min temperature gradient to 280 °C for 25 min. The injector temperature was 280 °C. Chromatography-grade helium was used as the carrier gas, with a flow of 2.7 mL/min. Mass spectra were recorded with an electron beam of 70 eV.

## 3. Results and Discussion

### 3.1. Reactivity of Tyrosinase towards Organosolv Lignin (OL)

The reactivity of tyrosinase towards lignin is debated. Lignans, the secondary metabolites resembling the dimeric units of lignin, generally showed inhibitory activity against tyrosinase [44,45,46], and the catalytic effect of tyrosinase in the oxidation of milled wood lignin was found to be negligible with respect to that of laccase and horseradish peroxidase [47]. On the other hand, tyrosinase from *Agaricus bisporus* was reported to cleave the 4-*O*-5 and Cα-Cβ bonds of some representative dimeric lignin model compounds (4-phenoxylphenol and guaiacyl glycerol-β-guaiacylether) [48]. For this reason, we decided to study the reactivity of tyrosinase from *Agaricus bisporus* towards organosolv lignin (OL) before the immobilization procedures, by treatment with tyrosinase in PBS under dioxygen atmosphere at room temperature to yield OL-tyr. After 24 h, the mixture was acidified and successively centrifuged to remove the supernatant. A sample of OL, treated under similar experimental conditions in the absence of tyrosinase, was used as the reference. The amount of characteristic OH groups in OL-tyr and OL was determined by quantitative ^31^P-NMR analysis [39]. Briefly, the samples were dissolved in pyridine/CDCl_3_ (300 µL; 1.6/1.0 *v*/*v*) under sonication conditions and phosphitylated in situ with 2-chloro-4,4,5,5-tetramethyl-1,3,2-dioxaphopholane. Table 1 shows the relevant experimental data collected. More specifically, aliphatic, condensed, guaiacylic, *para*-hydroxy phenyl, and acidic lignin OH groups were measured. Data are the averages of three phosphitylation experiments. The ^31^P-NMR data showed a modest decrease of the total phenolic OH and of the total OH groups after the enzyme treatment (11% and 9%, respectively), confirming the low reactivity of tyrosinase towards lignin.

Structural modifications in OL-tyr were further evaluated by Gel Permeation Chromatography analysis (GPC) of the residue. Table 2 reports the weight average molecular weight (M_w_), the number average molecular weight (M_n_), and the polydispersity (M_w_/M_n_) of OL-tyr compared to parent OL (the original GPC analysis is reported in Figure S1). Tyrosinase treatment induced an appreciable increase in the M_w_ and M_n_ values.

Thus, in our hand, tyrosinase showed a low reactivity towards OL, affording a modest decrease in the total amount of phenolic OH, associated with a modest, although significant, increase in the weight average molecular weight of the polymer, probably as a consequence of oxidative coupling processes [49].

### 3.2. Immobilization Procedures

#### 3.2.1. Encapsulation and Direct Adsorption Procedures

**Scheme 1:**
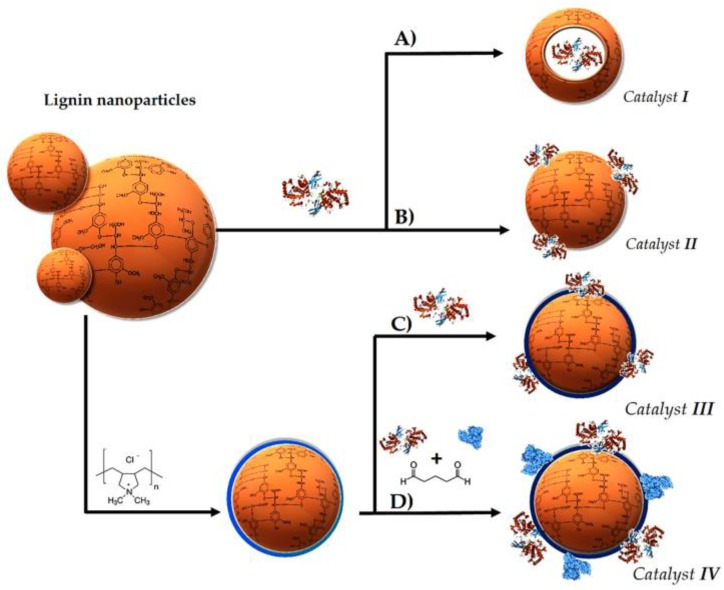
Schematic representation for the preparation of catalysts **I**–**IV**.

**Figure 1:**
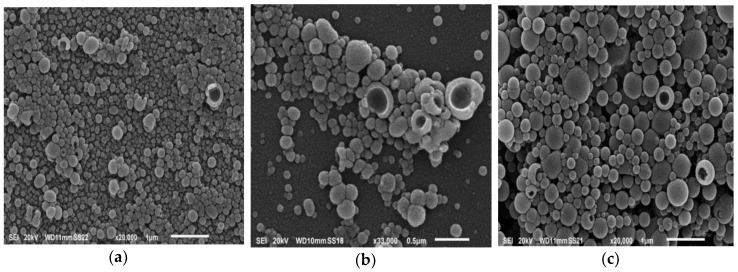
SEM images of catalyst **I** and catalyst **II**. (**a**) General overview of catalyst **I**; (**b**) Selected magnification of catalyst **I**; (**c**) General overview of catalyst **II**.

Alkali Lignin nanoparticles have been previously reported as containers for drug delivery systems [50]. The encapsulation procedure consisted of the formation of nanoparticles by slow solvent exchange in the presence of the bioactive principle to be immobilized, such as resveratrol, sorafenib, benzazulene, and ibuprofen [51,52]. Lignin nanoparticles showed high an antioxidant protective effect, successfully releasing the bioactive principle in the cell. A similar procedure was used in the encapsulation of dyes [53]. We decided to apply this procedure for the encapsulation of tyrosinase in OL nanoparticles. During the slow exchange of the solvent, the lignin molecules interacted together to close the nanoparticles, with tyrosinase being entrapped inside their empty core cavity. At the end, the encapsulated tyrosinase system (catalyst **I**) was recovered by centrifugation and washed to remove the excess of the enzyme (Scheme 1, pathway A). Scanning electron microscopy (SEM) images of catalyst **I** are reported in Figure 1.

**Figure 2:**
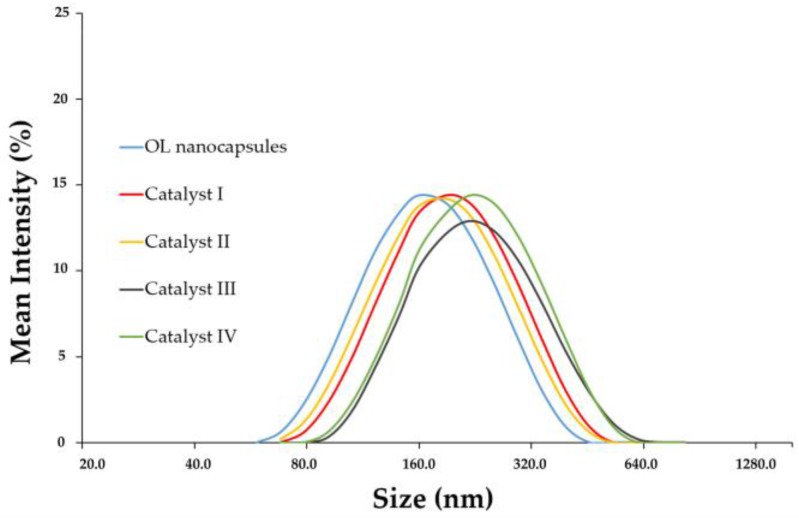
Representative particle diameter distributions of OL nanocapsules and Catalyst **I**–**IV**.

**Figure 3:**
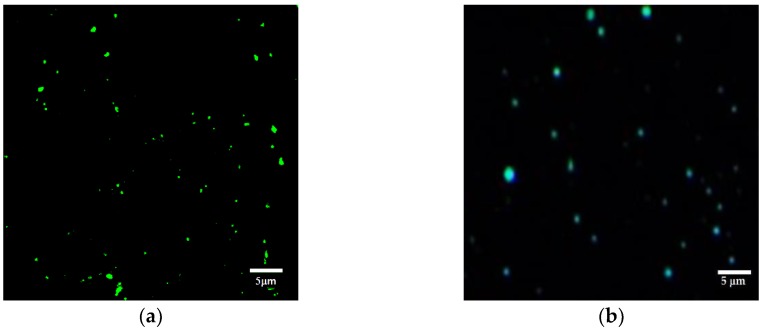
Confocal images of (**a**) Catalyst **I**; (**b**) Catalyst **II**.

**Figure 4:**
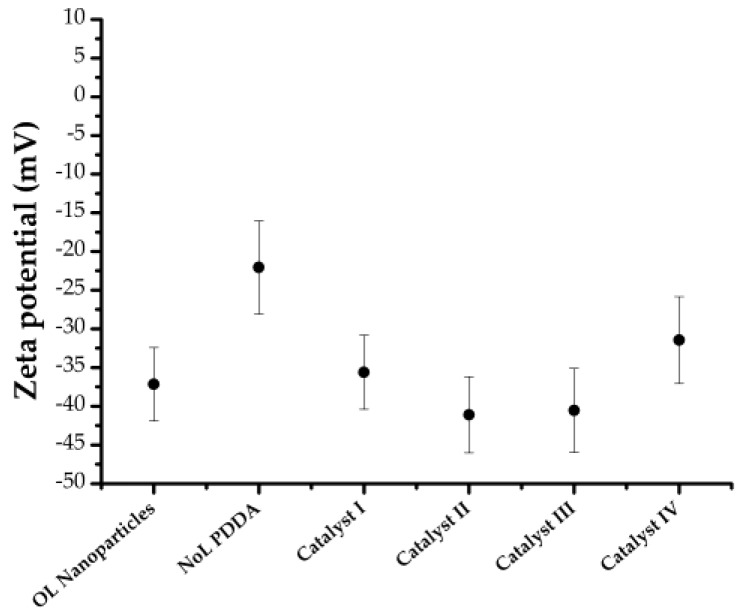
Zeta potential values of OL nanoparticles, OL PDDA nanoparticles, and catalysts **I**–**IV**.

The SEM image of catalyst **I** showed spheres of a regular shape (Figure 1, panel a) with an average diameter of 0.19 ± 0.01 µm, as evaluated b Dinamic Light Scattering (DLS) analysis (Figure 2). Fragmented particles were characterized by the presence of an empty cavity (Figure 1, panel b). The presence of the enzyme in catalyst **I** was observed by confocal analysis using a tyrosinase previously marked with Fluorescein isothiocyanate (FITC) [54]. As reported in Figure 3 (panel a), catalyst **I** showed the expected green fluorescence signal inside nanoparticles. The entrapment of tyrosinase was further confirmed by measuring the value of the Zeta potential of the particle surface, which was found to be very similar to the value obtained for the parent OL nanoparticles (Figure 4).

As an alternative, catalyst **II** was prepared by the direct addition of tyrosinase on preformed OL nanoparticles (Scheme 1, pathway B). Figure 1 (panel c) reports the SEM image of particles of catalyst **II**, characterized by an average diameter of 0.20 ± 0.01 µm (Figure 2). Catalyst **I** and catalyst **II** showed a similar morphology to SEM analysis. The confocal image of catalyst **II** (Figure 3, panel b), performed in the presence of the marked enzyme, showed a relatively weak green fluorescence signal only on the surface of nanoparticles (the background azure emission of the core being associated with the known auto-fluorescence of lignin [55]). Moreover, the Zeta potential of catalyst **II** varied with respect to parent OL nanoparticles (Figure 4). These data confirmed that the adsorption of tyrosinase was limited to the surface of the nanoparticles.

#### 3.2.2. Layer-By-Layer Immobilization Procedure

**Figure 5:**
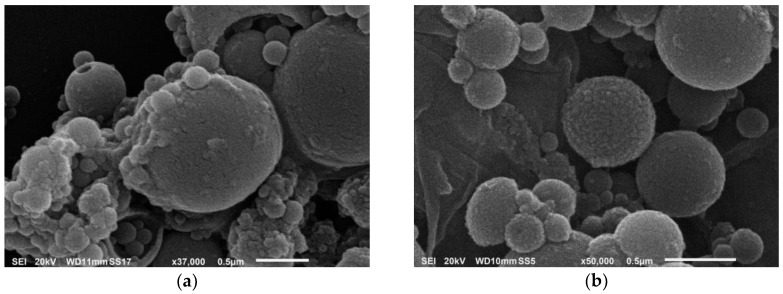
SEM images of catalyst **III** in the first panel (**a**) and of catalyst **IV** in the second panel (**b**).

The LbL procedure, based on the consecutive deposition of alternatively charged polyelectrolytes onto an appropriate support, protects enzymes from denaturing agents and allows regulation of the permeability towards small substrates, which can cross the multilayer and react with the catalytic site [56]. The use of the LBL procedure for the immobilization of tyrosinase from *Agaricus bisporus* on Multiwalled Carbon Nanotubes (MWCNTs) has been previously reported to increase the activity of the enzyme [29]. Since lignin nanoparticles are characterized by a negative value in terms of the Zeta potential (−42 mV at pH 4.2) [11], so we started with the deposition of a first positively charged layer of polydiallyldimethylammonium chloride (PDDA), able to efficiently interact with the lignin surface by cation–π interactions [57], to yield OL/PDDA nanoparticles. The deposition of the PDDA layer was monitored by UV-visible analysis [41], following the appearance of a typical peak at 276 nm, which corresponds to the shift of the characteristic PDDA B-band after adsorption on lignin (Figure S2). This result was further confirmed by the Zeta potential analysis (Figure 4), with the system reaching the value of −22 mV. Catalyst **III** was finally obtained by adding tyrosinase to OL/PDDA nanoparticles (Scheme 1, pathway C). Scanning electron microscopy (SEM) images of catalyst **III** (Figure 5, panel a) showed regular spherical particles with a wrinkled surface and an average diameter of 0.22 ± 0.02 µm (Figure 2). Catalyst **III** showed a Zeta potential value of −40 mV (Figure 4).

Successively, catalyst **IV** was prepared by applying the glutaraldehyde (GA)-assisted co-immobilization of tyrosinase in the presence of Bovine Serum Albumin (BSA) (Scheme 1, pathway D) [58]. As a general trend, the immobilization efficiency of tyrosinase is dependent upon the number of enzyme molecules loaded on the selected surface. The enzyme strives for the greatest surface coverage, leading to conformational changes that could influence the activity. BSA performs as an inert protein with an isoelectric point close to that of tyrosinase, to partially reduce the amount of surface area available for the enzyme, while GA forms a protective three-dimensional network [59]. Figure 5 (panel b) reports the SEM image of catalyst **IV**. The surface of catalyst **IV** appeared as a rough aggregate of small clumps with an average diameter of 0.22 ± 0.02 µm (Figure 2) and a Zeta potential value of −32 mV, significantly different from that previously measured for catalyst **III**. The Zeta potential analysis performed during the preparation of catalysts **III** and **IV** unambiguously confirmed the deposition of the tyrosinase layer on the initial OL/PDDA nanoparticles template.

### 3.3. Activity Parameters of Catalysts ***I***–***IV***

The Tyrosinase (Tyr) activity was determined by the dopachrome assay [60] following the oxidation of l-tyrosine at 475 nm, with the enzyme unit being defined in terms of the increase in absorbance of 10^−3^ unit/min at 25 °C in 0.1 M phosphate buffer (pH 7.0). The activity, activity yield, and immobilization yield, were evaluated as reported in Equations (1)–(3), respectively:

Activity (units/mg) = Ux/Wsupport(1)

Activity yield (%) = [Ux/(Ua − Ur)] × 100(2)

Immobilization yield (%) = [(Ua − Ur)/Ua] × 100(3)

where Ux is the activity (units) of the immobilized enzyme, Wsupport is the weight of the support (mg), Ua is the total activity (units) of the enzyme added to the solution, and Ur is the residual activity (units) in the washing solutions. As reported in Table 3, catalysts **III**–**IV** showed an immobilization yield value higher than that of catalysts **I**–**II**, suggesting a better performance of the LbL procedure in the immobilization of tyrosinase with respect to encapsulation and direct adsorption processes (entries 3–4 versus entries 1–2). The activity yield followed a similar trend. Note that catalyst **IV** was characterized by the highest activity yield value, confirming the beneficial role of BSA and GA in the retention of the tyrosinase catalytic performance after the immobilization process. The activity of catalyst **II**–**IV** was comparable to that previously reported for tyrosinase nanocatalysts [29]. The storage stability of the most active catalyst **IV**, as a selected sample, was evaluated by measuring the activity of the enzyme after 10 days of storage time in PBS solution at 5 °C under nitrogen atmosphere. Under these experimental conditions, catalyst **IV** completely retained its activity (Table 3, entry 4). These data were confirmed by ^31^P-NMR analysis of the lignin component after the storage, which showed any appreciable change in the total amount of OH groups.

### 3.4. Cyclic Voltammetry Analysis

**Figure 6:**
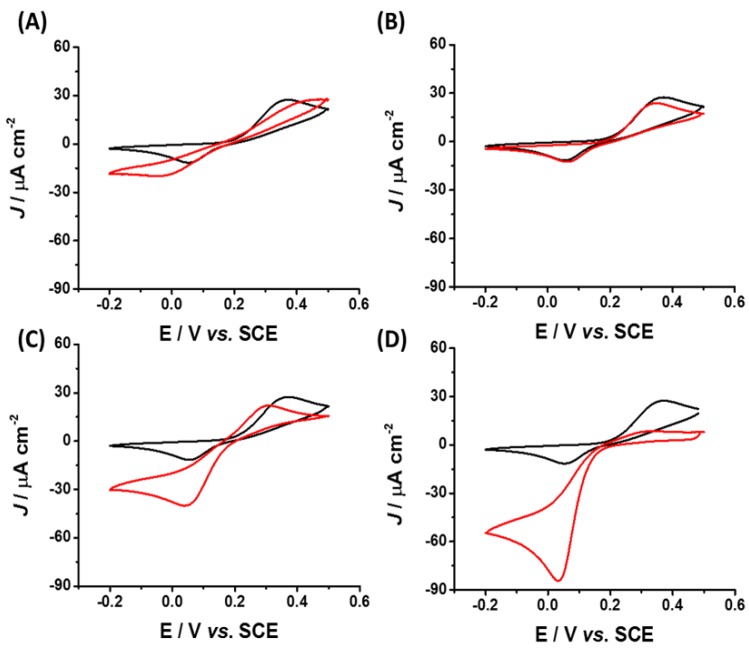
Cyclic voltammograms of OL nanocapsules (black line, **A**–**D**), Catalyst I (red line, (**A**)), Catalyst II (red line, (**B**)), Catalyst III (red line, (**C**)), and Catalyst IV (red line, (**D**)) performed in 250 µM catechol solution (50 mM PBS buffer pH 6.5 + 100 mM KCl) at a scan rate of 5 mV s^−1^.

Cyclic voltammograms (CVs) of catalysts **I**–**IV** were recorded at 25 °C in comparison with parent OL nanoparticles as a reference. The analyses were carried out in 250 µM catechol solution (PBS buffer, 50 mM; pH 6.5), in the presence of KCl (100 mM) as the supporting electrolyte. Irrespective of the catalysts studied, the electrocatalytic wave in CVs started in the correspondence of the formal potential (E^0′^) of catechol (0.190 V; measured versus Saturated Calomel Electrode SCE). Data are reported in Figure 6 (panels A–D; catalysts are marked by a red line, while the reference is reported as black line). As a general pattern, the CVs confirmed the previously observed trend for the activity parameters (Table 1). In particular, the CV of catalyst **I** (Figure 6, panel a) showed a well-defined electrocatalytic wave starting from 0.190 V vs. SCE and rising up to −20 µA cm^−2^ at −0.2 V vs. SCE. This data indicated that tyrosinase also retained an appreciable electrocatalytic activity in the encapsulation state. Catalyst **II** (Figure 6, panel b) was characterized by a weak electrocatalytic responsiveness (electrocatalytic wave from 0.190 V vs. SCE to −4 µA cm^−2^ at −0.2 V vs. SCE), in accordance with the low activity yield previously measured (Table 3, entry 2). Afterwards, the electrocatalytic responsiveness of catalyst **III** was found to be increased (electrocatalytic wave from 0.190 V vs. SCE to −30 µA cm^−2^ at −0.2 V vs. SCE). Finally, catalyst **IV** showed the highest electrocatalytic wave compared to the other catalysts, starting from 0.190 V vs. SCE to −56 µA cm^−2^ at −0.2 vs. SCE.

### 3.5. Kinetic Properties of Catalysts ***I***–***IV*** and Synthesis of Catechol Derivatives

Catalysts **I**–**IV** were characterized for their kinetic properties using l-Tyrosine (in the range 0.33–0.1 mM) as the substrate and plotting data to a double reciprocal Lineweaver–Burk plot (Table 4). Data for native Tyrosinase are also reported as a reference (Table 4, entry 1). Irrespective of the procedures used for the immobilization, V_max_ decreased and K_m_ increased for the supported tyrosinase, which led to a partial reduction in the catalytic efficiency with respect to free enzymes. Similar trends of K_m_ and V_max_ values have been reported for tyrosinase immobilized on other carriers, with them being attributed to mass transfer limitation processes [61] due to the restriction on the mobility of the enzyme after the immobilization procedure [62].

Catalysts **III**–**IV** showed a higher catalytic efficiency than catalysts **I**–**II** (Table 4, entries 4–5 versus entries 2–3), confirming the beneficial role of the LbL procedure. Note that catalyst **I** was more active than catalyst **II** (Table 4, entry 2 versus entry 3), probably as a consequence of conformational changes occurring when the enzyme was directly adsorbed on the surface of OL nanoparticles.

**Scheme 2:**
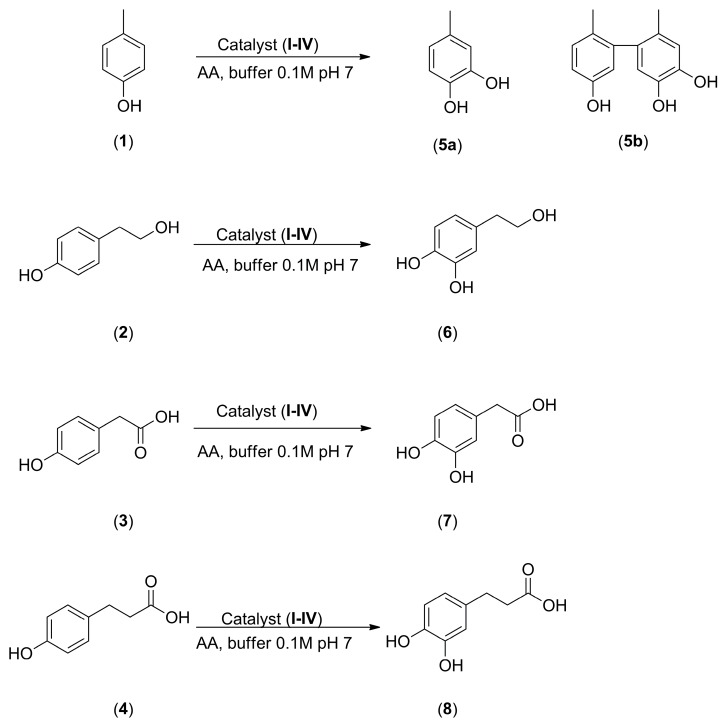
Oxidation of phenols **1**–**4** with catalysts **I**–**IV**.

The K_m_ and V_max_ values measured for catalysts **I**–**IV** were of the same order of magnitude as those recently reported for the tyrosinase crude extract of desert truffle (*Terfezia leonis* Tul.) after immobilization into a sol-gel silica layered matrix [43]. In this latter case, catalyst **IV** showed a K_m_ value (0.25 mM) lower than that observed after the sol-gel encapsulation procedure. Slightly lower values of K_m_ were also observed for catalyst **IV** with respect to the tyrosynase-silicate-Nafion system [63] and tyrosinase titania sol-gel films [64], respectively. The synthetic versatility of catalysts **I**–**IV** was then evaluated in the oxidation of a panel of selected phenols **1**–**4** (Scheme 2) to obtain the corresponding catechol derivatives **5**–**8**, including bioactive hydroxytyrosol **6** [65], and 3,4-dihydroxydihydrocinnamic acid **8** [66].

Catechols are characterized by several biological activities and are well recognized as antioxidant compounds [67]. The biotechnological potential of tyrosinase in the synthesis of catechols has received a great degree of attention because they are difficult to obtain under environmentally friendly conditions by means of traditional chemical procedures [68]. Briefly, phenols **1**–**4**, catalysts **I**–**IV** (540 units), and ascorbic acid AA (2.0 equiv) were stirred in PBS (0.1 M, pH 7.0, 5.0 mL) at room temperature, under a dioxygen atmosphere (O_2_). Ascorbic acid was used as an internal reducing agent to avoid the polymerization of the quinone intermediates [69]. As a general trend, catalysts **I**–**IV** were able to oxidize phenols to corresponding catechols from a moderate to high yield. No oxidation was observed in the presence of OL alone. Irrespective of the experimental conditions, catalysts **III** and **IV** were more reactive than catalyst **II**, affording the desired catechols in acceptable yields.

The oxidations were very selective, with catechol derivatives being the only isolated products besides the unreacted substrate. In the case of catalyst **I**, a lower selectivity was observed, since catechols were only detected at a low yield with respect to the total conversion of substrate (Table 5, entry 2–5). The low mass balance was probably due to over-oxidation processes, as confirmed by the isolation of a product of oxidative coupling, compound **5b**, in the oxidation of *para*-cresol **1** (Table 5, entry 2). Moreover, the low yield observed in the oxidation of phenol **3** (Table 5, entries 11–15) was probably ascribable to the known inhibitory activity reported for this compound against tyrosinase [70]. Recycling experiments proceeded with success in the oxidation of phenol **1**, as a selected example. After the first oxidation, catalysts **I**–**IV** were recovered by centrifugation, washed, and reused with fresh substrate. As shown in Table 6, catalysts **III**–**IV** were used for at least five cycles with only a slight decrease of activity. Otherwise, catalysts **I**–**II** lost ca. 50% of their original activity, suggesting the easier deactivation of tyrosinase in the absence of the LbL system.

Activity parameters (Table 3), cyclic voltammetry analyses (Figure 6), and kinetic properties (Table 4), clearly highlighted the best performance of the LbL procedure with respect to encapsulation and direct surface adsorption of tyrosinase. This trend was further confirmed by reactivity data (Table 5) from the oxidation of phenols, with the LbL-based catalysts **III**–**IV** being the most reactive systems. Moreover, the use of BSA as an inert protein spacer and GA reticulation further increased the activity of immobilized tyrosinase in catalyst **IV**, demonstrating the key role of conformational changes in the retention of the enzyme activity [71]. In accordance with previously reported data, the increased activity of LbL catalysts with respect to other systems might be explained on the basis of the electrostatic stabilization of the enzyme by interaction with the positive-charged polyelectrolyte counterpart [72], as well as the benign hydrophobic pocket interactions between the adjacent layers [73]. The lower immobilization yields of catalysts **I**–**II** (Table 3) might be reasonably attributed to the electrostatic repulsion between tyrosinase and the OL nanoparticles bearing a similar negative charge at the operative pH. Most probably, the electrostatic repulsions are also responsible for the lower activity yield of catalysts **I**–**II** (Table 3) as a consequence of conformational changes involving the assessment of negatively charged amino acid residues of the enzyme with respect to the inner or outside surface of the support, respectively [19].

## 4. Conclusions

Organosolv lignin was shown to be an efficient and renewable support for the immobilization of tyrosinase. Phosphorus-based Nuclear Magnetic Resonance ^31^P-NMR and GPC analysis demonstrated the stability of lignin towards the phenoloxidase activity of tyrosinase, with the low increase of the weight average molecular weight observed after the tyrosinase treatment being a consequence of quinone induced cross-reticulation processes. Notably, the cross-reticulation has been reported as a favorable process for the stabilization of the oxidative catalytic activity of lignin organized in vesicular reverse micelles [74]. Among the different procedures applied for the preparation of the catalysts, the layer-by-layer deposition of PDDA on pre-formed lignin nanoparticles, followed by the deposition of tyrosinase and BSA, yielded the most active system for the oxidation of phenols to corresponding catechols. The layer-by-layer procedure performed better than the direct adsorption of tyrosinase on lignin nanoparticles, and as an alternative, encapsulation of the enzyme inside the cavity of lignin nanoparticles. Microscopy analysis (SEM and confocal analysis), Zeta potential measurements, and cyclic voltammetry confirmed the immobilization of the enzyme, furnishing data about the effect of the immobilization procedure on the chemical and physical properties of the catalyst. Again, after the layer-by-layer deposition, tyrosinase showed the highest electrocatalytic responsiveness and the highest activity and kinetic parameters. Probably, undesired conformational changes of the enzyme, occurring during the direct adsorption or encapsulation processes, are responsible for the lowering of the catalytic efficacy of tyrosinase.

## Figures and Tables

**Table 1:** Functional OH distribution (mmol/g) for parent OL and for OL-tyr after treatment with tyrosinase ^a^.

Sample	Carboxylic Acids	Aliphatic -OH	Condensed -OH	Phenolic-OH ^b^			
				Para-Hydroxy Phenyl -OH	Guaiacyl -OH	Total Phenolic -OH	Total Group -OH
**OL**	0.22	0.99	1.95	0.24	0.68	2.87	4.08
**OL-tyr**	0.19	0.97	1.79	0.17	0.59	2.55	3.71

^a^ Quantitative ^31^P-NMR spectra were performed after dissolution of the sample in pyridine/CDCl_3_ (300 µL; 1.6/1.0 *v*/*v*) under sonication conditions and phosphitylation with 2-chloro-4,4,5,5-tetramethyl-1,3,2-dioxaphopholane. OL: original sample of organosolv lignin; OL-tyr: organosolv lignin after treatment with tyrosinase. ^b^ Maximum standard deviation: 2 × 10^−2^ mmol/g. Maximum error: 1 × 10^−2^ mmol/g.

**Table 2:** M_w_, M_n_, and M_w_/M_n_ of OL tyr compared with the parent OL ^a^.

Sample	M_w_	M_n_	M_w_/M_n_
OL	154.670	15.104	10.2
OL-tyr	202.828	18.803	18.8

^a^ Analyses were repeated in triplicate.

**Table 3:** Activity parameters of catalysts **I**–**IV**.

Entry	Catalyst	Immobilization Yield %	Activity Yield % ^a^	Activity (Units/mg) ^b^
1	**I**	69	34	6.8
2	**II**	71	31	6.2
3	**III**	90	42	8.4
4	**IV**	87	58	11.6 (11.3) ^c^

^a^ The activity of native tyrosinase, evaluated by the dopachrome method, was 900 units/mg of enzyme. ^b^ The enzyme loading refers to the percentage of the initial tyrosinase supported on the catalyst. ^c^ Activity of catalyst **IV** after 10 days of storage time at 5 °C under nitrogen atmosphere.

**Table 4:** Kinetic parameters of Tyr and catalysts **I**–**IV** ^a, b^.

Entry	Catalyst	K_m_ (mM)	V_max_ × 10^−3^ (∆Abs min µg_enzyme_)^−1^	V_max_/K_m_ (×10^−3^)
1	Tyr	0.18	6.02	33.44
2	**I**	0.51	3.40	6.77
3	**II**	0.67	1.14	1.70
4	**III**	0.37	3.89	10.51
5	**IV**	0.25	4.10	16.40

^a^ V_max_ represents the maximum rate achieved by the system, at a saturating substrate concentration. The Michaelis constant K_m_ is the substrate concentration at which the reaction rate is half of V_max_. ^b^ All experiments were conducted in triplicate using free and immobilized tyrosinase. Average errors in kinetic parameters were 2–4% for K_m_ and 1–3% for V_max_. l-Tyrosine was used as a substrate.

**Table 5:** Oxidation of Phenols **1**–**4** with Native Tyrosinase and Catalysts **I**–**IV** ^a^.

Entry	Catalyst	Substrate	Product(s)	% Conversion	% Yield
**1**	Tyr	1	**5a**	99	98
**2**	**I**	1	**5a[5b]**	91	20 [18]
**3**	**II**	1	**5a**	40	39
**4**	**III**	1	**5a**	59	59
**5**	**IV**	1	**5a**	75	75
**6**	Tyr	2	**6**	96	96
**7**	**I**	2	**6**	35	25
**8**	**II**	2	**6**	20	18
**9**	**III**	2	**6**	38	37
**10**	**IV**	2	**6**	46	46
**11**	Tyr	3	**7**	77	77
**12**	**I**	3	**7**	18	7
**13**	**II**	3	**7**	15	15
**14**	**III**	3	**7**	21	20
**15**	**IV**	3	**7**	28	28
**16**	Tyr	4	**8**	78	78
**17**	**I**	4	**8**	34	12
**18**	**II**	4	**8**	30	30
**19**	**III**	4	**8**	57	56
**20**	**IV**	4	**8**	67	65

^a^ The oxidation was performed on 0.02 mmol of substrate with the appropriate catalyst (540 units) in PBS (0.1 M pH 7) at 25 °C under O_2_ atmosphere for 24 h.

**Table 6:** Recycling of catalysts **I**–**IV** in the oxidation of *para*-cresol **1** ^a^.

Entry	Run	Catalyst I (Yield %)	Catalyst I (Yield %)	Catalyst III (Yield %)	Catalyst IV (Yield %)
**1**	**1**	20	39	59	75
**2**	**2**	20	39	59	75
**3**	**3**	15	32	51	70
**4**	**4**	11	27	46	67
**5**	**5**	9	21	42	61

^a^ Reusability is expressed as the yield in % of catechol **5a** obtained by the oxidation of 1 with catalyst **I**–**IV**.

## Supplementary Materials

The following are available online at http://www.mdpi.com/2079-4991/8/6/438/s1, Figure S1: Gel Permeation Chromatography (GPC) of lignin Organosolv (OL black line) and after the treatment with tyrosinase (OL-tyr grey line), Figure S2: The UV-vis absorption spectra of OL nanoparticles coated with PDDA.
