# Supplementary material for: Functionalized Tyrosinase-Lignin Nanoparticles as Sustainable Catalysts for the Oxidation of Phenols
Source: Nanomaterials (Basel). 2018 Jun 15;8(6):438. doi: 10.3390/nano8060438 (PMC6027214; doi:10.3390/nano8060438)
Supplement: Supplementary file 1 [file nanomaterials-08-00438-s001.pdf]

Functionalized Tyrosinase-Lignin Nanoparticles as Sustainable Catalysts for the Oxidation of phenols

Eliana Capecchi[1], Davide Piccinino[1], Ines Delfino[1], Paolo Bollella[2], Riccarda Antiochia[2], Raffaele Saladino[1, *]

[1] Department of Biological and Ecological Sciences, University of Tuscia, Via S. Camillo de Lellis snc, 01100 Viterbo, Italy; eliana.capecchi@studenti.unitus.it; d.piccinino@unitus.it; delfino@unitus.it.

[2] Department of Chemistry and Drug Technologies, Sapienza University of Rome P.le Aldo Moro 5, 00185 Rome, Italy; paolo.bollella@uniroma1.it**;** riccarda.antiochia@uniroma1.it.

*Correspondence: saladino@unitus.it; Tel. 39 0761 357284:

Structural modifications in OL-tyr evaluated by Gel Permeation Chromatography analysis (GPC)

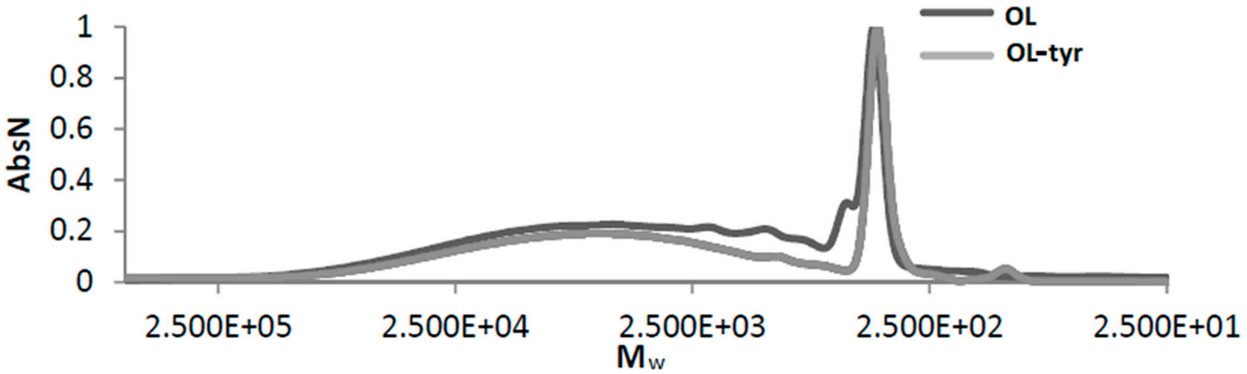

Figure S1. Gel Permeation Chromatography (GPC) of lignin Organosolv (OL black line) and after the treatment with tyrosinase (OL-tyr grey line).

The UV-vis absorption spectra of OL nanoparticles and OL-PDDA nanoparticles

The qualitative determination of the interaction between OL nanoparticles and OL-PDDA nanoparticles was demonstrated with UV-vis absorption measurements. Figure S2 show the UV-vis spectra of PDDA (Brown line), OL nanoparticles (Blue line) and OL-PDDA nanoparticles (Grey line). Adsorption peak at 276 nm corresponds to the shift of the characteristic B band of PDDA when adsorbed on lignin, this result has been previously reported and confirm the deposition of the PDDA layer on OL nanoparticles [1].

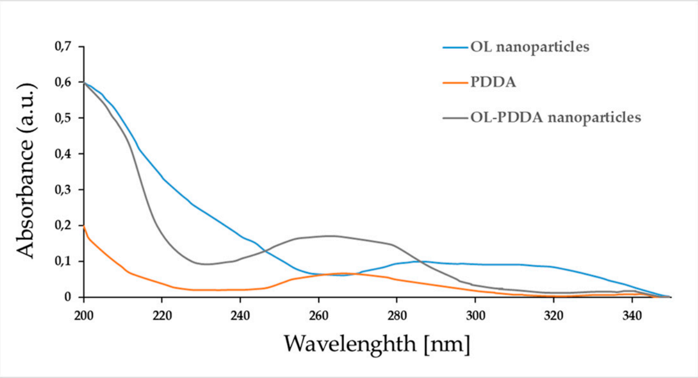

Figure S2. The UV-vis absorption spectra of OL nanoparticles coated with PDDA.

[1] Jiang, C.; He, H.; Jiang, H.; Ma, L.; Jia, D. M. Nano-lignin filled natural rubber composites: Preparation and characterization. *Express Polym. Lett.* **2013**, *7* 480-493; doi:10.3144/expresspolymlett.2013.44.
